# Estimating carbon and water footprints associated with commercial milk formula production and use: development and implications of the Green Feeding Climate Action Tool

**DOI:** 10.3389/fnut.2024.1371036

**Published:** 2024-06-13

**Authors:** Julie P. Smith, Bindi Borg, Tuan T. Nguyen, Alessandro Iellamo, Andini Pramono, Roger Mathisen

**Affiliations:** ^1^National Centre for Epidemiology and Population Health, College of Health and Medicine, The Australian National University, Canberra, ACT, Australia; ^2^Crawford School of Public Policy, College of Asia and the Pacific, The Australian National University, Canberra, ACT, Australia; ^3^Alive & Thrive, Global Nutrition, FHI 360, Hanoi, Vietnam; ^4^Crisis Response, FHI 360, Washington, DC, United States

**Keywords:** breastfeeding, commercial milk formula (CMF), greenhouse gases (GHG), carbon footprint, water footprint, first-food system, innovation, sustainable development goals (SDGs)

## Abstract

Carbon offset frameworks like the UN Clean Development Mechanism (CDM) have largely overlooked interventions involving food, health, and care systems, including breastfeeding. The innovative Green Feeding Climate Action Tool (GFT) assesses the environmental impact of commercial milk formula (CMF) use, and advocates for breastfeeding support interventions as legitimate carbon offsets. This paper provides an overview of the GFT’s development, key features, and potential uses. The offline and online GFT were developed using the DMADV methodology (Define, Measure, Analyze, Design, Verify). The GFT reveals that the production and use of CMF by infants under 6 months results in annual global greenhouse gas (GHG) emissions of between 5.9 and 7.5 billion kg CO_2_ eq. and consumes 2,562.5 billion liters of water. As a national example, in India, one of the world’s most populous countries, CMF consumption requires 250.6 billion liters of water and results in GHG emissions ranging from 579 to 737 million kg CO_2_ eq. annually, despite the country’s high breastfeeding prevalence among infants under 6 months. The GFT mainly draws on data for low- and middle-income countries (LMICs), as many high-income countries (HICs) do not collect suitable data for such calculations. Despite poor official data on breastfeeding practices in HICs, GFT users can input their own data from smaller-scale surveys or their best estimates. The GFT also offers the capability to estimate and compare baseline with counterfactual scenarios, such as for interventions or policy changes that improve breastfeeding practices. In conclusion, the GFT is an important innovation to quantify CMF’s environmental impact and highlight the significance of breastfeeding for planetary as well as human health. Women’s contributions to environmental preservation through breastfeeding should be recognized, and breastfeeding interventions and policies should be funded as legitimate carbon offsets. The GFT quantifies CMF’s carbon and water footprints and facilitates financing breastfeeding support as a carbon offset initiative under CDM funding facilities.

## Highlights

- Breastfeeding women’s contribution to environmental protection, including mitigating GHG emissions and conserving water resources, should be acknowledged and documented.
- The carbon and water footprints of the CMF industry can and should be measured, and the environmental harms mitigated by breastfeeding acknowledged.
- The Green Feeding Climate Action Tool estimates the carbon and water footprints of CMF production and use.
- Policies and interventions that better enable breastfeeding should be measured and funded as carbon offsets.

## 1 Introduction

Climate change poses immense risks to human health and well-being. With 3.6 billion people already living in areas highly susceptible to climate change, 250,000 additional deaths are expected each year from 2030 to 2050 due to malnutrition, malaria, diarrhea and heatwaves alone (1). Low- and middle-income countries (LMICs) will be least able to cope, and infants and young children are particularly vulnerable. Since 2015, governments have formally acknowledged that urgent action is needed to address climate change risks and reduce greenhouse gas (GHG) emissions (1).

Most attention is on climate change caused by the GHG emissions from the use of fossil fuels. However, food production and consumption is another major driver of environmental harms, generating one-third of all GHGs (2). The contemporary food system also generates high levels of land and habitat degradation, depletion and contamination of fresh and marine water resources, and waste production. The recent accelerating global transition to unhealthy diets high in meat, dairy, and ultra-processed foods put both human and planetary health at risk (3). The livestock industry is responsible for almost one-fifth of global GHG emissions, and the dairy industry makes up approximately one-fourth of that (4). Furthermore, over three-quarters of the world’s water is consumed in agricultural production (5). In addition, unnecessary and unhealthy processed foods that are high in fat, sugar, salt, and calories, but low in nutrients also contribute significantly to water scarcity (5).

Rapid and radical transformation of the global food system will be needed if the Sustainable Development Goals (SDGs) and global climate goals are to be met (6). Agricultural policies such as the European Union’s Common Agricultural Policy for 2023–2027 aim to nurture sustainable and environmentally friendly food production (7). For over a decade, there have been calls to link national dietary guidelines with sustainability concerns (8). “First-food systems” that provide food for infants and young children (9) also need to be scrutinized for their climate change implications, especially as the global baby-food industry strengthens its attempts to increase commercial milk formula (CMF) sales, thereby undermining breastfeeding (10). While food systems differ across the planet, the highly evolved “first-food system,” namely breastfeeding, is potentially universally accessible at local level. For optimal nutrition and health, exclusive breastfeeding is recommended for the first 6 months of an infant’s life, continuing to 2 years and beyond along with safe and suitable complementary foods. Globally, less than half of all infants under 6 months (47%) are exclusively breastfed during the previous 24 h, and available data suggests CMF use correlates with rising *per capita* GDP and declining continued breastfeeding rates (11). Yet the sustainable development implications of infant and young child diets, and in particular the carbon and water footprint of CMF products (most of which are manufactured from dairy milk powder), are rarely considered in discussions on approaches to climate change risk mitigation.

In the context of climate change, global heating, and increasingly frequent disasters and emergencies, infants and young children are exposed to greater risks such as changing patterns of infectious disease, heat stress, and malnutrition due to food supply chain disruptions. The adaptive composition of human breastmilk reduces the harms of heat stress in infants and strengthens the immune system against known and novel infections. Breastfeeding and breastmilk availability helps protect against malnutrition during food shortages (11).

Thus breastfeeding is a mitigating and adaptive response, and high breastfeeding prevalence strengthens population resilience (12). Conversely, CMF has high environmental impacts, is maladaptive to climate change risks, is vulnerable to supply interruptions, and reduces resilience of populations of infants and young children during disasters and emergencies.

### 1.1 Commercial food products for infants and young children and their carbon and water footprints

The CMF industry is growing rapidly, driven by factors such as urbanization, medicalization of childbirth, rising maternal labor force participation, and aggressive marketing (9, 10).

A growing literature now identifies the environmental impacts of declining breastfeeding prevalence, with evidence accumulating over the past two decades (13–15). Breastfeeding NGOs, notably the International Baby Food Network (IBFAN) and the Geneva Infant Feeding Association (GIFA), highlighted the environmental costs of CMF feeding over 30 years ago (13, 16). Two recent studies further detailed several environmental impacts of commercial baby food and milk formula products in addition to GHG emissions and water consumption (17, 18). These include chemical and biological pollution and contamination of water with resultant marine and freshwater eutrophication; soil degradation, acidification and depletion; deforestation and biodiversity loss; and antimicrobial resistance. The solid waste that arises from packaging of CMF is significant. In the United States alone, it is estimated that 86,000 tons of metal and 364,000 tons of paper from CMF packaging is added to landfills each year, without considering the waste generated in transportation (19). Bottles and teats which are necessary for feeding CMF also have a high environmental impact.

CMF production and consumption involve multiple agricultural and industrial steps, with dairy milk production being a major contributor to its environmental impact (17, 20). This impact is primarily via dairy milk production’s carbon and water footprints (18, 21). CMF production consumes a large amount of water. The total water required to produce a kilogram of CMF is approximately 5,000 L or more (17, 22).

Growth in CMF markets is therefore a move away from SDG goals for healthy and sustainable food systems.

### 1.2 The importance of breastfeeding for nutrition, health, and sustainability

Contrasting with CMF, breastfeeding is the biologically normal way to feed human infants and young children. Early initiation and exclusive breastfeeding for 6 months provides the necessary nutrition and immunological protection for optimal health, growth, and development; conversely, inadequate breastfeeding is responsible for a significant proportion of infant morbidity and mortality, in both LMICs and HICS (23–30). Breastfeeding has long been considered a “Double Duty Nutrition Action” because it addresses both childhood undernutrition and overnutrition (31, 32). Breastfeeding also reduces mothers risk of non-communicable diseases, including postnatal depression, breast and ovarian cancer, diabetes, and cardiovascular disease (23, 33). With its wide-ranging protective effects for maternal, child, and lifelong health, breastfeeding reduces cost burdens on health systems (32, 34, 35). The World Health Organisation (WHO) 2030 Global Nutrition Targets aim for 70% of infants to be exclusively breastfed to 6 months of age, and 70% to continue breastfeeding for 2 years or more (36).

With growing evidence on the large GHG emission impacts of current infant and young child feeding practices, it has been argued that breastfeeding is in fact a “Triple Duty Action” (37, 38) addressing not only undernutrition and overnutrition, but also sustainability (39). Breastfeeding uses few resources and produces zero or minimal waste. Breastfeeding addresses climate policy pillars of mitigation, adaptation, and resilience (11, 12). It has been argued that improving protection, support, and promotion of breastfeeding should be a global priority in addressing current unsustainable food systems (37, 38).

In addition to its significant contribution to sustainable consumption and production, combatting climate change, and conserving marine and terrestrial ecosystems (SDGs 12, 13, 14, and 15), increased breastfeeding and reduced CMF use is fundamental to the broader sustainable development agenda (40) and the achievement of the SDGs (41–43). The direct contribution of breastfeeding to Goals 2 and 3 (ending hunger and improving nutrition, food security, health, and wellbeing) are clear. Breastfeeding also contributes to improved educational attainment (Goal 4) and employment achievement, as well as economic growth (Goal 8), thus contributing to the reduction of poverty and inequality (Goals 1 and 10). Breastfeeding and actions to support it contribute to gender equality including reproductive and employment rights (Goal 5). In keeping with the holistic and indivisible nature of the SDGs, the World Alliance for Breastfeeding Action (WABA) has articulated how breastfeeding is relevant to the achievement of all of the SDGs (44).

However, there has been only slow progress toward optimal infant and young child feeding practices in recent decades, and few countries are on target (45). While exclusive breastfeeding rates have increased slightly to around 47%, continued breastfeeding rates are declining (46).

### 1.3 Existing online tools

The innovative Green Feeding Climate Action Tool (GFT) complements three existing nutrition tools which focus on breastfeeding that have been developed in the past decade. These are:

- the 2013 World Breastfeeding Costing Initiative (the WBCi Costing Tool) which estimates the funding required to implement interventions that support and promote optimal infant and young child feeding (47).
- the Cost of Not Breastfeeding (CNB) Tool, launched in 2019, which calculates the potential health, human capital, and economic costs of not breastfeeding as country-level costs (34, 48).
- the Mothers’ Milk Tool (launched in 2022), which calculates the volume and value of breastmilk produced by an individual mother or on a national level and, by corollary, the economic production loss incurred due to suboptimal breastfeeding practices (49).

These existing tools quantify the health and economic losses due to not breastfeeding but do not address the environmental impacts.

### 1.4 Aims

The GFT seeks to fill this gap by calculating carbon and water footprints from CMF products used as substitutes for breastfeeding.

The objectives in developing the GFT were to create an online and downloadable tool that could:

- estimate the carbon and water footprints of CMF,
- be easily used by policymakers, influencers, project managers, environmentalists, researchers, national accountants, statisticians, and breastfeeding advocates, and
- complement evidence-based advocacy for interventions that can mitigate climate change and enable breastfeeding.

This paper aims to describe how the GFT was developed, highlight some of the key features of the tool, and consider potential uses of the tool.

## 2 Methods

**Figure 1:**
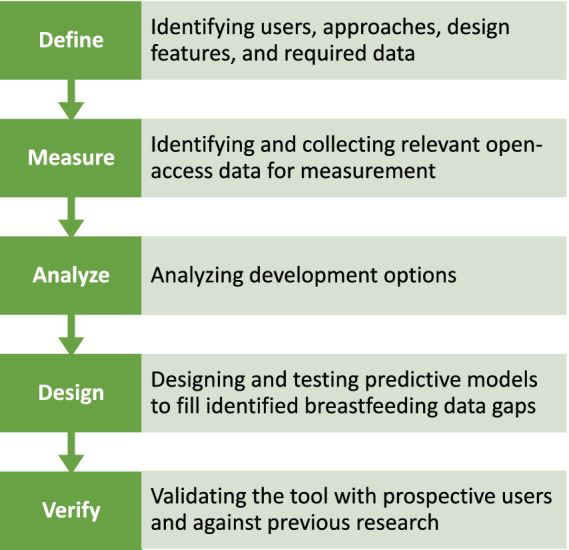
DMADV diagram for the development of the GFT (50, 51).

The development process of the GFT follows a structured approach known as DMADV (Define, Measure, Analyze, Design, and Verify), depicted in Figure 1. The approach utilizes the steps of defining project goals, acquiring relevant data, analyzing design options, creating a prototype, and verifying the tool’s functionality with users (50, 51).

### 2.1 Step 1. Define (goal, output, literature and existing tools, users, uses, approaches, design features, data required)

The primary goal in creating the GFT was to calculate the carbon and water footprints of CMF for infants under 6 months at national levels. The output envisaged consisted of estimates of the amount of GHG created and water used for all infants (exclusively breastfed (EBF), predominant breastfed (PBF, breastmilk and water only), non-breastfed (assuming using CMF), and partially breastfed (the remaining, assuming using CMF in the country selected by the user).

The GFT would be used for calculating the environmental effects of breastfeeding promotion, support, and protection interventions, or devising policies that improve breastfeeding practices. Quantifying the carbon and water footprint in this way provides data for budget calculations and policy considerations. This can enable policy makers and advocates to consider comparative options for investments and decisions that aim to address environmental issues, including those related to infant feeding. It was expected that the GFT would confirm the importance of policies, strategies, and interventions that protect, support, and promote breastfeeding.

One of the main uses envisaged for the GFT is to advocate for including breastfeeding promotion, support, and protection interventions as fundable projects under carbon offset schemes and financing mechanisms such as the United Nations Clean Development Mechanism (CDM). The CDM transitioned to the Article 6.4 mechanism of the Paris Agreement at the UN Climate Change Conference in Glasgow (COP26) in 2021 (52). However, for ease of reference, we continue to use the term CDM. The CDM requires a methodology based on a standard and replicable procedure of calculating CMF emissions at baseline, compared to a counterfactual scenario following an intervention (53, 54).

The developers of the GFT expected that users would include government policy makers, as well as non-government organizations (NGOs) and international agencies concerned with environmental issues. The experience of similar tools emphasized the importance of a design that was flexible enough to meet users’ needs for customization while also allowing for updates and improvements based on user feedback. Online and offline versions of the GFT were envisaged.

Existing tools were reviewed for insight into the users and uses, approaches, design features, and data that would be needed for the GFT. Data is needed on the number of infants under 6 months, and on the prevalence of CMF use. The small body of literature recognizing and estimating the carbon and water footprints of CMF was reviewed to identify information on key parameters to be used for GFT estimates. Literature that examined the resource inputs and environmental impacts of CMF was identified and examined as far back as 1989 (13, 16). The focus of this literature was on CMF producing and consuming countries in Europe, America, and Asia. A number of studies made empirical estimates of the carbon footprint per kilogram (kg) of CMF (14, 15, 18, 20), while two studies estimated the water footprint (14, 17). The literature review demonstrated that, while the environmental impact of CMF at various stages of the product life cycle was recognized and was feasible to measure, what was lacking was a flexible tool to use this information for estimates of country-level impacts for advocacy and to inform decision making.

### 2.2 Step 2. Measurement

In this step, the GFT developers identified the best sources of open access data. The criteria were that data were able to be regularly updated and consistently available to allow for efficient automatic updating of the GFT with limited time or investment. While it is possible to estimate CMF consumption from industry sales data, such information is expensive and not easily updated. Instead, it was determined that UNICEF data on the number of infants who are partly or completely CMF fed could be accessed and used for this purpose. Data required included population estimates for infants under 6 months, and rates of exclusive or predominant, partial, and non-breastfed. UNICEF data on births provided approximate estimates of the 0 to under 6 months population in any year. The UNICEF infant and young child feeding (IYCF) database (55) provides data that is preloaded in the GFT for 80 LMICs that conduct Multiple Indicator Cluster Surveys (MICS) and Demographic and Health Surveys (DHS). For countries that do not conduct MICS and DHS, the GFT provides a solution whereby users can enter their own data on EBF plus PBF, partial breastfed, and non-breastfed from other sources. It is important to note that the accuracy of the GFT calculations is directly contingent upon the availability and reliability of the data manually entered by the user.

For the purposes of GFT calculations, the GFT “exclusively and predominantly breastfed.” All other infants are considered to be partially or completely CMF fed (partially breastfed and non-breastfed), although it is acknowledged that some such infants will receive animal milk or other foods. Non-breastfed infants were assumed to require approximately 20 kg of CMF powder for infants under 6 months, based on UNHCR protocols which also includes stock waste. “Partially breastfed” infants are assumed to receive one-third of their nourishment from CMF (that is, requiring 6.7 kg of CMF powder for infants under 6 months), in addition to breastfeeding.

The GFT utilizes current data from recent lifecycle analyses of the carbon footprint of CMF per kg of powdered product. In one study, the total estimated CO_2_ eq. GHG emissions of each kg of CMF powder over the full product life cycle is between 11 and 14 kg, with most of this (68–82%) attributable to raw milk production (20). The results of this study, which is used for the GFT estimates of carbon footprints, suggest that around 200 kg of GHG eq. emissions were generated from the 20 kg CMF needed to feed an infant from birth to 6 months. In a 2021 study, CMF feeding of infants for 6 months generated a somewhat larger carbon footprint of between 226 and 288 kg CO_2_ or approximately 7.5–9.6 kg CO_2_/month (17).

Hence, the GFT use the following formula (Equation 1) to calculate GHG emissions associated with the use of CMF among infants under 6 months:


(1) $$\begin{array}{l} GHG\;\mathrm{emissions}\;\left(\mathrm{kg}\;{CO}_{2}\;Eq.\right)=\\ \;[6.7\;{\mathrm{kg}}^{*}\;\left(\mathrm{number}\;\mathrm{of}\;\mathrm{infants}\;\mathrm{who}\;\mathrm{are}\;\mathrm{partially}\;\mathrm{breastfed}\right)\\ \;+20\;{\mathrm{kg}}^{*}\;\left(\mathrm{number}\;\mathrm{of}\;\mathrm{infants}\;\mathrm{who}\;\mathrm{are}\;non‐\mathrm{breastfed}\right){]}^{*}\;\\ \;GHG\;\mathrm{emissions}\;\mathrm{per}\;\mathrm{kg}\;\mathrm{of}\;CMF\;\mathrm{powder} \end{array}$$

In which, the GHG emission per kg of CMF powder is 11 kg for the lower estimation and 14 kg for the upper estimation.

CMF also places high demands on scarce water resources. The GFT utilizes the most recent estimates of the water footprint of CMF production and consumption. Water use in production includes drinking water for dairy cattle and for producing feed, and cleaning and cooling of equipment and facilities. A recent study calculated that 699 L of “blue” water use (that is, water harvested for household or industrial use) are required to produce 1 kilogram of CMF (the average can of formula is 800 g) (17). However, when green (rain) and grey (run-off) water are included, the true water footprint for every kg of formula is much higher, ranging from 4,700 to 7,430 L (17). The consumption and use of CMF also requires water. For each kilogram of CMF prepared hygienically, reconstitution requires about seven liters and bottle washing and sterilization requires about 56 L (17). The bare minimum—for example, in emergency settings—is three liters of water a day (13). Further details on how the tool makes its calculations are in the online brief and downloadable instructions for the offline tool.

### 2.3 Step 3. Analysis of tool design options

The GFT was created with two main components: a “baseline” module and a customizable module. The basic module has a dashboard that shows the carbon and water footprint estimations of a selected country using the most up-to-date pre-loaded data. It was necessary to overcome the limitations of data available for pre-loading by allowing the user to enter their own data on births and infant feeding practices. Hence, an “enter own data” functionality provides flexibility for users to input data for previous years. It also allows this data to be entered for sub-national levels if breastfeeding and birth data is available at this level.

To meet the aim of developing a methodology suitable for calculating carbon offsets in alignment with CDM requirements for certifying interventions, the GFT needed to give users flexible options to create scenarios and compare baseline estimates of carbon footprints with estimates for different infant feeding practices scenarios. Thus, an important feature of GFT design is a customizable module for calculating carbon and water footprints for counterfactual scenarios. This allows the GFT to meet the requirements of the CDM methodologies, by allowing the carbon offset of activities or projects to be calculated and certified as a carbon offset generating a specific amount of carbon credits. CDM methodologies include requirements to estimate “additionality,” namely the difference between emissions at baseline and the emissions levels after the intervention is in place. This required that in the GFT, users can input data to calculate customized counterfactual scenarios such as the expected GHG reduction impacts of breastfeeding policies, programs, or project interventions on GHG emissions and water resource use. Conversely, the GFT has the flexibility for the user to calculate the GHG implications of scenarios involving declines in exclusive or partial breastfeeding, such as in contemplation of the future “extinction” of exclusive breastfeeding in a country (56). Examples of real-world policy scenarios were used to test the functionality and are described below.

CDM methodologies also require accounting for leakage (an indirect effect of emission reduction interventions that lead to a rise in emissions elsewhere). Consideration was given to whether estimates should adjust for changes in maternal diet during lactation, and for the child spacing effects of breastfeeding. Additional food intake is commonly recommended for lactating women. Some studies estimate that lactating women require approximately 500 kcal/day (20). However, this may not be universally required to ensure adequate maternal nutrition (57–59). Breastfeeding exclusively for 6 months may generate between 123 and 162 kg CO_2_ eq. GHG emissions (17), depending on the composition of the additional dietary intake, i.e., animal-source versus plant-based foods (18).

A further relevant consideration is the effect of breastfeeding on child spacing. Fifty percent more births would be expected in the absence of breastfeeding in countries where continued breastfeeding is prevalent (23, 60, 61). Population growth is a major driver of GHG emissions but accounting for these emissions is a contentious issue (62, 63).

It was decided that the GFT would not automatically adjust for maternal diet, or for child spacing effects of breastfeeding on carbon and water footprints. Key studies found that for most countries and dietary scenarios, exclusive breastfeeding has a lower carbon footprint than CMF even after assuming all mothers needed additional intake (18, 20). Whether breastfeeding mothers require additional food intake depends on individual nutritional status, stored fat reserves from pregnancy, and changes in activity levels. Also, the ethical position is taken that women’s diets should always meet their nutritional needs including during pregnancy and breastfeeding; the tool is not designed for measuring the GHG impacts of addressing nutritional deficits.

However, the GFT allows the user to make separate approximations of the GHG impact for a nutrition intervention for undernourished women through a separate module within the tool. This is done by applying the counterfactual function to input data such as for a breastfeeding intervention which includes enhanced maternal nutrition. Using this functionality, the user can choose one of two common dietary patterns. Based on the findings of research on diets in Vietnam and Norway (18), these are (1) a mainly plant-based diet (assuming GHG of 69 kg CO_2_e for 6-month period), or (2) a mixed plant and animal-based diet (GHG of 218 kg CO_2_e for 6-month period).

The GFT does not make calculations for children aged 6–36 months using CMF, follow-on formulas, or growing up milk products. This would require detail on complementary feeding practices which is not available. Furthermore, adjusting the calculations for variations in local diets and complementary feeding practices for young children across the ages 6–36 months would be too complex. However, it is important to note that the estimated GHG emissions for CMF products sold for this age category is substantial, and in a study of six countries in Asia, it contributed to around three-quarters of all CMF related GHG emissions (37).

### 2.4 Step 4. GFT development

#### 2.4.1 Development of the offline version

**Figure 2:**
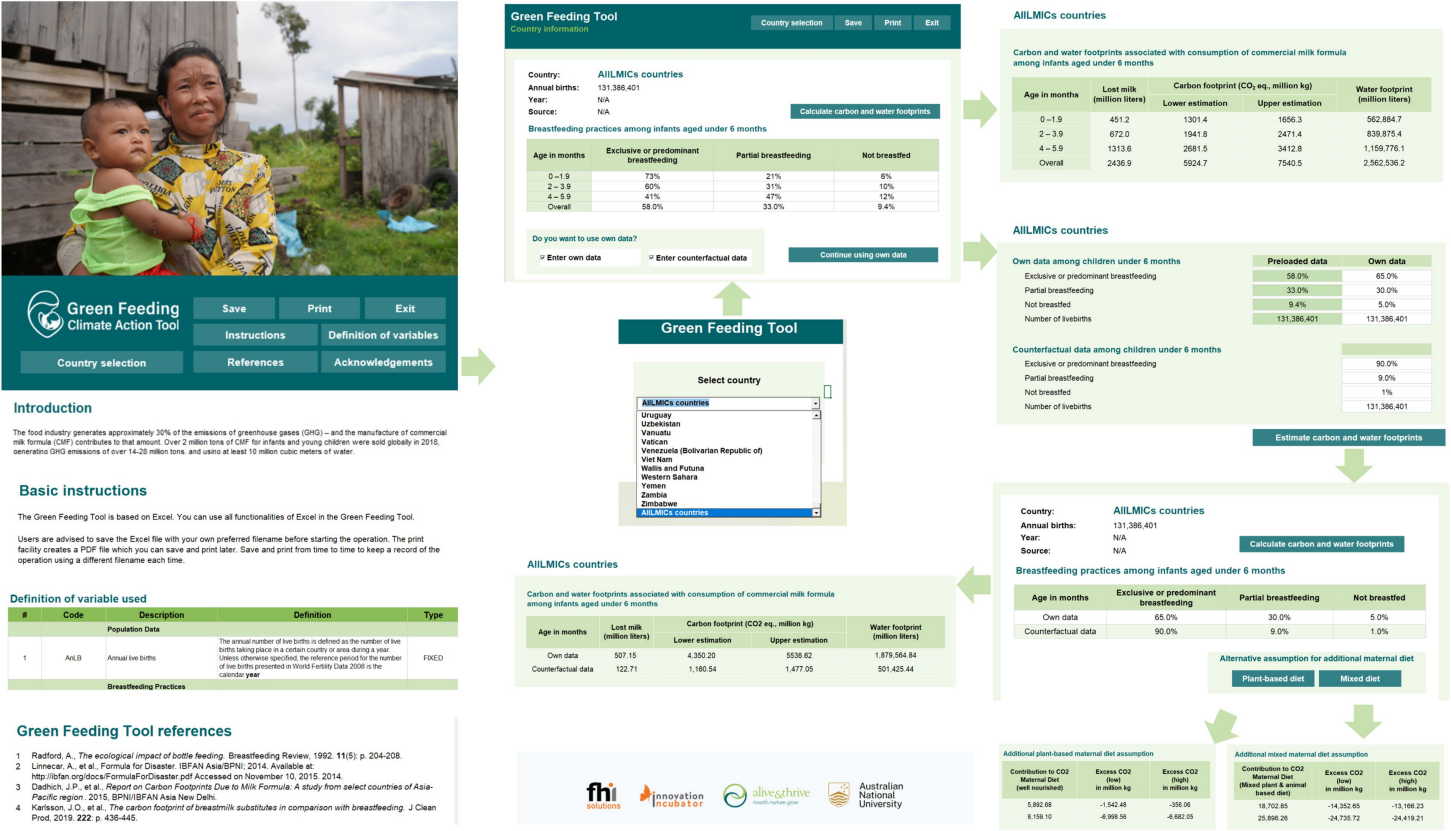
Key functions of the offline version of the GFT. Authors created this figure using snapshots of the GFT offline (https://greenfeedingtool.org). The human identifiable images are licensed for personal, business, or commercial purposes.

During 2022, the offline tool was developed in Microsoft Excel using the approaches and parameters identified (Figure 2). Functions for calculating carbon and water footprints and presenting results for baseline and counterfactual calculations were created. The design was tested using data for several individual countries, and then country datasets were uploaded for testing in the final functioning offline tool. Readers can click the “Definition of variables” button in the GFT^1^ to see descriptions and definitions of the variables and the formulas used to calculate results.

After the user selects a country with preloaded data, the country calculator provides a table with rows showing the number of infants under 6 months, categorized as 0–1 months, 2–3 months, 4–5 months, and under 6 months. The columns show the breastfeeding practices, namely exclusively and predominantly breastfed, partially breastfed, and non-breastfed. Cells contain percentages of each group. This screen also indicates the source and year of the data. The following screen shows a low and high calculation for the carbon footprint (using 11 kg CO_2_ eq. GHG emissions and 14 kg CO_2_ respectively) and a water footprint. In addition to country-specific data, the GFT includes the possibility to make a calculation for all LMICs.

The user may choose to load their own data, for example, when data has not been pre-loaded because the country does not collect or share that data with the UNICEF IYCF database. This is common for HICs that have poor data and do not follow the globally recommended indicators for assessing IYCF. For example, below we calculate an example for China using data on breastfeeding practices from a survey (64) that provided estimates of the prevalence of exclusive, predominant, and partial breastfeeding under 6 months.

Alternatively, some users may want to calculate projected changes in CMF, and subsequently, changes in carbon and water footprints, to reflect the effects of policies, strategies, and interventions that may lead to increases or decreases in breastfeeding. These users can select the option to “Enter own data” or “Enter counterfactual” respectively. They will be asked to enter their data and the estimated carbon and water footprints will be calculated with the inputted data. To illustrate this functionality, in the Results section, we show the estimated carbon and water footprints of an increase in paid maternity leave in Canada in 2008 which was found to have increased exclusive breastfeeding (65).

Another example of this kind is provided below, using available country data on exclusive breastfeeding rates, is to use the GFT functionality for inputting “own data” and estimating a “counterfactual” result, in a scenario where the Baby-Friendly Hospital Initiative (BFHI)/Ten Steps to Successful Breastfeeding is implemented in Indonesia. Data from a 2022 before-after study of a hospital in Hong Kong (66) showed that implementation of the BFHI might be expected to raise exclusive breastfeeding rates by 15%, any breastfeeding rates by 25%, and reduce exclusive formula-feeding rates (non-breastfed) by 25%. A calculation using the own data and counterfactual functionality of the GFT can be made (at country level, or if data on annual births is available, for a particular province or hospital), again on the assumption that the prevalence of predominant breastfeeding is zero.

Finally, the GFT was used to illustrate an “extinction” scenario below, in which breastfeeding from under 6 months is entirely displaced by exclusive CMF feeding. Such a scenario has been postulated in a futurist analysis (56), and can also be done for individual countries using the GFT counterfactual functionality.

#### 2.4.2 Development of the online version

**Figure 3:**
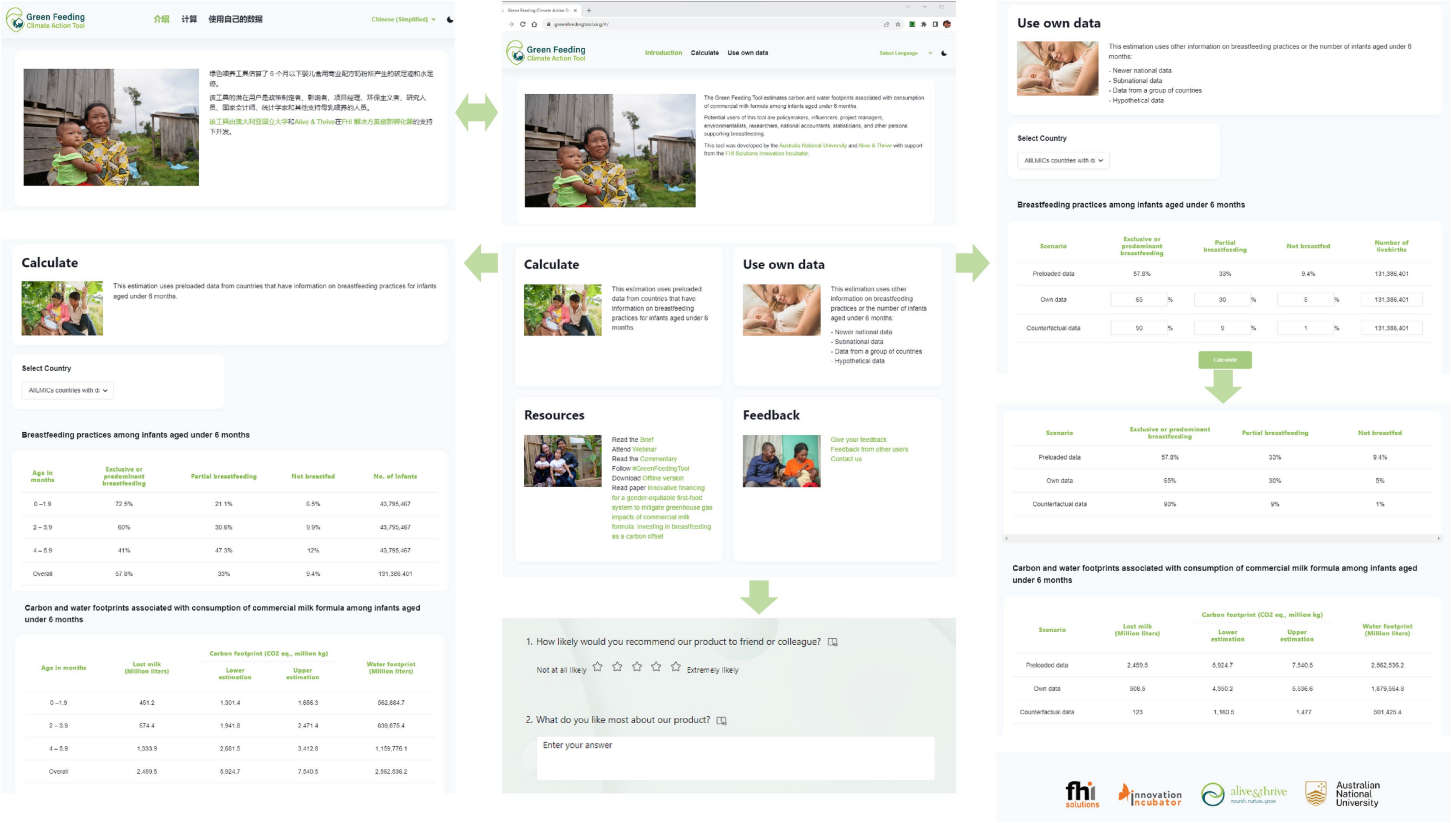
Key functions of the online version of the GFT. Authors created this figure using snapshots from https://greenfeedingtool.org. The human identifiable images are licensed for personal, business, or commercial purposes.

The offline version of the GFT was launched in a webinar on 5th June 2023 with 290 participants from 64 countries. While participants had various backgrounds, most were nutritionists, pediatricians, lactation consultants, and midwives. The webinar recording is available for viewing (see https://nceph.anu.edu.au/research/projects/green-feeding-tool). The online version of the GFT was then developed and is available in all major languages. Refer to Figure 3 for key functions and flow of the GFT online.

Building upon the offline version, a local Information Technology (IT) consultant supported the development of the GFT online version. A core team member engaged in discussions with this consultant to outline the essential components, functions of the GFT, and the expectations, as well as provide background information regarding the estimations. The IT consultant then proceeded to develop the online tool. A core team member cross-checked the findings of the online version with those from the offline version. Slight modifications of the wording in the GFT facilitate translation across languages. The improved wording was incorporated into the offline version.

### 2.5 Step 5. GFT validation

Validation occurred at multiple points in the development process, as described below. Once the Excel version was functioning, it was initially piloted in a small working group. Improvements were made based on the feedback from that group. The GFT was then circulated to a larger group from different global regions that included potential users from diverse fields including IYCF and environment. Invitations to test were sent out on 6th April 2023. Testers were asked to provide feedback on the GFT functions and ease of use, and on potential reliability and utility of the results. A total of 14 respondents, all female, from 11 countries, tested the GFT. Testers were advocates for IYCF and environment with diverse backgrounds including government bodies and NGOs.

The additional improvements were made based on the input of this testing group and were mostly to presentation. Branding guidelines from FHI 360 were used to ensure consistency in presentation. Suggestions were also made on future functions that could be added to the GFT.

The GFT results were also compared with country estimates in five published studies to better understand sources of variance in estimates and compare GFT estimates with studies using similar or different methodologies. Comparison required some standardization even where a common methodology is used. For example, the GFT calculations use preloaded data on infant feeding to generate estimates of CMF use, but most country studies use Euromonitor data on sales of a variety of CMF products. Different assumptions are made about GHG emissions per kg of CMF, or about CMF requirements for the 6-month period. Since some of the studies were conducted, infant feeding practices have changed considerably. Also, approximations of “exclusive and predominant,” “partial,” and “no” breastfeeding, prevalence must be used in these country comparisons due to lack of authoritative data in suitable form. Supplementary Table S1 summarizes this standardization and testing of GFT results.

The GFT also provides estimates of “lost milk,” which can be interpreted here as the potential gain from higher breastfeeding rates, or alternatively, as a simple measure of the extent of vulnerability of a country’s infant and young child population to climate change risks. As more granular country data on feeding practices for infants under 6 months is used in the GFT than was possible in the Mothers’ Milk Tool which covers children 0–36 months (49), calculating the “lost milk” output allows cross checking for consistency. The MMT calculates milk quantities very conservatively based on “any” breastfeeding rather than the more precise “exclusive and predominant,” “partial,” and “no” breastfeeding, which is the basis for GFT calculations. Hence, MMT estimates of “lost milk” will be consistently higher than results for the same country using the GFT.

Feedback continues to be collected using the online form or email from the GFT, as well as through communication with potential users during dissemination events, conferences, and meetings, ensuring ongoing improvement of the GFT.

## 3 Results

The GFT calculates the annual GHG emissions and water use that could be mitigated by policies which reduce CMF use among infants under 6 months, or the additional carbon and water footprints that would result from further expansion of infant formula markets.

For example, the GFT can calculate the carbon and water footprints of 80 (mostly LMIC) countries for which suitable data are available. By selecting “All LMICs countries” in the GFT, we calculate that the aggregate consumption of CMF by infants under 6 months in these countries results in annual emissions ranging from around 5,925 to 7,541 million kg of CO_2_ equivalent GHG emissions and consumes 2,562,500 million liters of water annually. These results can be seen in the first row of Table 1.

**Table 1:** Carbon and water footprints associated with CMF use among infants under 6 months, GFT estimates from pre-loaded data for 20 most populous countries.

Pop. rank	Country	Population (million)^1^	Annual live birth (million)	Lost milk (million liters)	Carbon footprint (CO_2_ eq., million kg)		Water footprint (million liters)
					Lower estimation	Upper estimation	
	LMIC countries		131.4	2,459.5	5,924.7	7,540.5	2,562,536.2
1	India	1428.6	22.6	226.0	579.3	737.3	250,572.5
2	China	1425.7	11.5	–	–	–	–
3	United States	340.0	3.7	–	–	–	–
4	Indonesia	277.5	4.4	96.1	213.8	272.1	92,460.0
5	Pakistan	240.5	6.1	99.0	261.9	333.3	113,281.7
6	Nigeria	223.8	7.4	65.3	198.4	252.5	85,802.2
7	Brazil	216.4	2.7	–	–	–	–
8	Bangladesh	173.0	3.0	22.5	66.9	85.2	28,952.6
9	Russia	144.4	1.4	–	–	–	–
10	Mexico	128.5	1.9	80.6	167.4	213.0	72,391.4
11	Ethiopia	126.5	3.8	44.1	102.6	130.5	44,356.9
12	Japan	123.3	0.8	–	–	–	–
13	Philippines	117.3	2.4	–	–	–	–
14	Egypt	112.7	2.4	37.7	95.6	121.7	41,356.9
15	DR Congo	102.3	3.8	35.4	103.2	131.4	44,644.1
16	Vietnam	98.9	1.5	21.2	63.8	81.2	27,603.5
17	Iran	89.2	1.2	–	–	–	–
18	Turkey	85.8	1.2	–	–	–	–
19	Germany	83.3	0.8	–	–	–	–
20	Thailand	71.8	0.7	29.0	56.5	71.9	24,449.9

^1^Population by Country (2023)—Worldometer (worldometers.info).

Of the world’s 20 most populous countries, 11 have data suitable for preloading into the GFT (Table 1). Using the GFT to select these countries, annual CO_2_ equivalent emissions from CMF use for infants under 6 months are calculated at around 579.3–737.3 million kg in India, 213.8–272.1 million kg in Indonesia, and 63.8–81.2 million kg in Vietnam. Annual water use from infant formula use was 250,572.5 million liters in India, 92,460 million liters in Indonesia, and 27,603.5 million liters in Vietnam.

For countries with limited data on breastfeeding practices, users can input their own data from smaller-scale surveys or their best estimates. Similarly, for countries where preloaded data on infant feeding practices is not available, the GFT allows users to input their own data. In the case of China, we used the available data to input a prevalence of exclusive and predominant breastfeeding under 6 months of 61.3% and partial breastfeeding of 32.2% (64). This generated an estimated carbon footprint of 437.4–556.7 million kg CO_2_ eq. and a water footprint of 189,000 million liters (Table 2). In a counterfactual but hypothetically feasible calculation for China, which assumed that policy or program interventions improved exclusive/predominant breastfeeding to 90%, the corresponding estimate of carbon footprint reduced to 118.4–150.7 million kg CO_2_ equivalent and a water footprint of 51,167 million liters.

**Table 2:** Using own data and counterfactual data to estimate GHG and water footprints in China.

	Breastfeeding practice (%)		Annual number of births	Lost milk (million liters)	Carbon footprint (CO_2_ eq., million kg)		Water footprint (million liters)
	Exclusive or predominant	Partial			Lower estimation	Upper estimation	
Preloaded data							
Data in 2018 (64)	61.3	32.2	11,501,936	53.6	437.4	556.7	189,000
Counterfactual	90.0	8.0	11,501,936	15.3	118.4	150.7	51,167
Difference	28.7	−24.2		−38.3	−319.0	−406.0	−137,833

The tool also offers the capability to estimate scenarios for specific policy changes which affect infant feeding practices such as an increase in paid maternity leave. In Canada in 2008, such a policy change was found to have increased exclusive breastfeeding from 23.1 to 31.5% (65). We input this data to calculate the environmental implications of this policy change. For this calculation, no data on predominant breastfeeding was available. Therefore, the prevalence of exclusive breastfeeding was input, predominant breastfeeding was assumed to be zero, and exclusive CMF feeding was assumed to reduce commensurately with the increase in exclusive/predominant breastfeeding. By reducing annual CMF consumption, this policy change is estimated to have decreased Canada’s annual GHG emissions by 3.9–4.9 million kg CO_2_ eq. and water consumption by 1,661.7 million liters a year (Table 3).

**Table 3:** Impact of paid maternity leave in Canada in 2008 on exclusive breastfeeding, CMF consumption and GHG emissions.

	Breastfeeding practice (%)		Annual number of births	Lost milk (million liters)	Carbon footprint (CO_2_ eq., million kg)		Water footprint (million liters)
	Exclusive or predominant	Partial			Lower estimation	Upper estimation	
Preloaded data							
Before 2008 (65)	23.1	51.3	373,864	5.3	35.2	44.8	15,204.8
After 2008 (65)	31.5	45.7	373,864	4.8	31.3	39.9	13,543.1
Difference	8.4	−5.6		−0.5	−3.9	−4.9	−1,661.7

To take another example, the 2018 Demographic Health Survey in Indonesia reported exclusive and partial breastfeeding rates of 58.2 and 29.9%, respectively (67), which we input into the GFT. Table 4 shows results comparing GHG and water footprints for the current situation compared to a universal BFHI/Ten Steps scenarios with 15 and 10 percentage point increases in EBF with potential coverage of BFHI in 10, 15, and 20% of births. For this calculation, the partial breastfeeding rate was estimated as two-thirds of the prevalence of not exclusively or predominantly breastfeeding, serving as the rate of preloaded data.

**Table 4:** Impact of BFHI intervention on GHG and water footprints in Indonesia.

	Breastfeeding practice (%)		Annual number of births	Lost milk (million liters)	Carbon footprint (CO_2_ eq., million kg)		Water footprint (million liters)
	Exclusive or predominant	Partial			Lower estimation	Upper estimation	
Preloaded data in 2018	58.2	29.9	4,435,250	96.1	213.8	272.1	92,460.0
Counterfactual							
15% increase in EBF and PBF rate	73.2	17.9	4,435,250	22.1	149.3	190.0	64,488.2
BFHI coverage 5%	73.2	17.9	221,763	1.1	7.5	9.5	3224.4
BFHI coverage 10%	73.2	17.9	443,525	2.2	14.9	19.0	6,448.8
BFHI coverage 15%	73.2	17.9	665,288	3.3	22.4	28.5	9,673.2
BFHI coverage 20%	73.2	17.9	887,050	4.4	29.9	38.0	12,897.6
10% increase in EBF and PBF rate	68.2	21.2	4,435,250	26.2	172.7	219.8	74,629.6
BFHI coverage 5%	68.2	21.2	221,763	1.3	8.6	11.0	3731.5
BFHI coverage 10%	68.2	21.2	443,525	2.6	17.3	22.0	7,463.0
BFHI coverage 15%	68.2	21.2	665,288	3.9	25.9	33.0	11,194.4
BFHI coverage 20%	68.2	21.2	887,050	5.2	34.5	44.0	14,925.9

As a contrast to the present-day GFT calculation for all LMICs, described in Table 1, we imagined an “extinction” scenario, in which all breastfeeding in LMICs is completely replaced by CMF feeding. In this scenario, as seen in Table 5, if breastfeeding were completely replaced by CMF feeding in these countries, the GFT calculated that the amount of GHG emissions and water consumed would increase current estimates fivefold. This would lead to increased annual CMF consumption, contributing to an increase in current global annual GHG emissions by 22,980.3–29,247.7 million kg CO_2_ eq. and water footprint of 9,926,266.8 million liters a year.

**Table 5:** Impact of an “extinction scenario” for breastfeeding in LMICs, and carbon and water footprints.

	Breastfeeding practice (%)		Annual number of births	Lost milk (million liters)	Carbon footprint (CO_2_ eq., million kg)		Water footprint (million liters)
	Exclusive or predominant	Not breastfeed			Lower estimation	Upper estimation	
Preloaded	57.8	9.4	131,386,401	2,459.5	5,924.7	7,540.5	2,562,536.2
Extinction	0	100	131,386,401	5,912.4	28,905.0	36,788.2	12,488,803.0
Difference	−57.8	90.6	0	3,452.9	22,980.3	29,247.7	9,926,266.8

## 4 Discussion

### 4.1 Key findings of the green feeding tool

The GFT provides important new information on the global and national environmental implications of infant feeding practices. It calculates that currently, the CMF used for infants under 6 months generates at least 6–8 billion kgs of CO_2_ eq. and uses over 2,562 billion liters of water each year.

The GFT has important functionalities that allow calculations of the GHG emission and water use impacts of policies and interventions that affect infant and young child feeding practices. These are illustrated for potential improvements in breastfeeding practices in China, a real-life policy change to paid maternity leave that occurred in Canada, a potential maternity services policy measure that is available in Indonesia, and a global “extinction” scenario involving CMF displacing all current exclusive or predominant breastfeeding in LMICs. Such a scenario—involving 29–37 billion kg of CO_2_ eq. GHG emissions and 12.5 billion liters of water use annually—remains an alarming possibility in the absence of strong actions by governments to protect, promote, and support breastfeeding.

Previous studies had indicated the environmental implications of CMF (13–15, 17, 18, 20–22). However, the innovative GFT allows for customized assessments of environmental impacts in a given country. The scale of impacts revealed by the GFT is substantial and highlights the environmental benefits and health co-benefits of investments in policies enabling breastfeeding. The results generated by the GFT can be interpreted by using comparative information from other sources such as the US Environmental Protection Agency Tool. For example, this shows that the current 7.5 billion kg of CO_2_ eq. GHG emissions in LMIC are equivalent to driving 19.3 billion miles in an average combustion engine vehicle while the water required (2,562 billion liters) could fill 819,840 Olympic swimming pools (69). Since national breastfeeding data are typically unavailable in HICs, calculations cannot be derived for these regions. However, users may utilize any accessible or hypothetical data to make estimations.

Since national breastfeeding data are typically unavailable in HICs, calculations cannot be derived for these regions. However, users may utilize any accessible or hypothetical data to make estimations.

### 4.2 Interventions to increase breastfeeding and decrease CMF

Interventions to promote, support, and protect breastfeeding and reduce CMF consumption are proven demand-side measures that can mitigate climate change (21). The GFT can be used to assess key environmental impact of these interventions, thereby providing a method of evaluating interventions that had hitherto not been implemented.

The costs and effectiveness of several such interventions were summarized in a recent systematic review (70). Peer counseling interventions increase exclusive breastfeeding by 48–90% (35, 71, 72). Comprehensive Baby Friendly Hospital Initiative (BFHI) implementation increases exclusive breastfeeding by 49% (35). Workplace support such as lactation rooms and breaks increase any breastfeeding up to 6 months by 25% (35). Media including social media to counteract industry marketing including “greenwashing” can increase early initiation of breastfeeding more than fivefold (35, 72). Implementing the WHO International Code of Marketing of Breastmilk Substitutes through legislation has minimal costs (35).

Paid maternity leave has been demonstrated to improve breastfeeding as well as maternal and child health outcomes (73), and it is now clear that paid maternity leave can improve environmental outcomes too (74). Any duration and level of paid maternity leave is associated with higher rates of exclusive breastfeeding (35, 72, 75). Where women are provided with 6 months of paid maternity leave, they are 30% more likely to exclusively breastfeed their infant from birth to 6 months of age (76). Conversely, reductions in paid maternity leave lead to reduced breastfeeding initiation and duration (77) and exclusive breastfeeding (78). When paid maternity leave was reduced in Norway, mothers who had the option took longer unpaid leave, including because they wanted to continue breastfeeding (79). Thus, increased CMF use and subsequent environmental harms should be acknowledged as a potential cost of policies that shorten maternal access to paid leave following childbirth.

### 4.3 Implications for national policy, programming, and budget decision making

The GFT provides new data on environmental footprints for policy, programming, and budget decision making, and is particularly powerful when used alongside the Cost of Not Breastfeeding Tool and the Mothers’ Milk Tool. In addition to the considerable human health costs of not breastfeeding (34), the innovative GFT makes it possible to quantify key environmental costs. When policy makers consider the financing of interventions to support, promote and protect breastfeeding (70), they will now be readily able to incorporate calculations of these environment impacts.

Similarly, the GFT can show the extent to which national breastfeeding strategies align with environmental concerns. The cost of supporting breastfeeding can be compared to the costs of other climate change mitigation, adaption, and resilience initiatives.

The GFT could be especially powerful if used in conjunction with a mapping and analysis of national and transnational stakeholders influencing relevant actions and policies in relation to breastfeeding and use of breastmilk substitutes, as has been done in other settings (80). This would be a useful area for future investigation.

### 4.4 Implications for global policy, programming, and budget decision-making

Global organizations, especially those financing health interventions, will be able to use the insights provided by GFT in the same way that nations can. Moreover, given the information that the GFT provides, it behooves agencies that are financing climate protection or operating carbon accounting schemes to consider breastfeeding as a fundable intervention. For example, an activity such as a project, program, or policy that results in higher breastfeeding rates might be counted as an “offset” to emissions from other activities (81). This could be part of funding schemes devised to provide financial incentives for investments in GHG emission reduction, to achieve global targets for reducing emissions and mitigating climate change.

At present, the food system as a source of GHG emissions is poorly recognized in mechanisms such as the CDM, Global Environment Facility, Green Climate Fund’s Adaptation and Readiness programs, and Just Energy Transition Partnerships, which provide carbon emission mitigation funds. The GFT provides the kind of data that would help to justify financing of investments in breastfeeding protection, support and promotion as a transformational climate change intervention with co-benefits for health. The World Health Organization’s Alliance for Transformative Action on Climate and Health (ATACH), which has committed to helping countries facilitate access to climate change funding for health (1, 82), could advocate for this. Such a move would address The Lancet’s call to break the silos and collaborate to address the global syndemic created by the combined pandemics of undernutrition, overnutrition, and climate change (39).

Carbon credits could in principle be claimed by CMF producer or consumer countries. It could be argued that a country producing CMF, by producing less, is reducing GHG emissions. In this paper, it is assumed that a country consuming less CMF due to its interventions to support breastfeeding, could claim credits for reducing its carbon footprint. However, production-phase policy interventions are also possible. Both production- and consumption-based estimates are available in existing scientific studies (20), and the parameters for per unit emissions used in the GFT mostly encompass this range.

### 4.5 Implications for food policy

The suite of carbon accounting products also includes national and international climate friendly guidelines and standards, (such as ISO14001), product labeling, and carbon tax (like sugar tax) on products. Including CMF products in standards for carbon footprints could be a useful avenue to explore for mitigating food system impacts (83). The GFT provides a way of calculating the environmental impact of CMF.

### 4.6 Implications for personal decision-making tools

Solutions to climate change cannot be left with individuals; responsibility necessarily lies mostly with governments and industry (84). Governments must take responsibility for creating policies and programs that make exclusive breastfeeding recommendations achievable in practice, to thereby reduce the necessity and consumer demand for breastmilk substitutes such as CMF.

However, a range of motivations affect women’s decisions to continue breastfeeding or introduce CMF, and the GFT provides data that could be used in carbon footprint calculators or in breastfeeding promotion to help raise awareness of environmental implications and influence individual decisions in response to breastfeeding challenges.

### 4.7 Strengths and limitations of the GFT

The GFT provides important new data on environmental implications of CMF use. A significant strength of the GFT is its simplicity and flexible functionality. It uses pre-loaded data that updates automatically, and has flexibility for adjustment to user needs, and to changing parameters as scientific evidence is updated.

The online availability and ease of use of the GFT makes it readily available to a variety of users across the world. It is intended to facilitate consideration of country investments in breastfeeding protection, promotion and support as a carbon offset. It has the flexibility to make calculations for counterfactual vs. baseline scenarios, in line with required methodologies to identify additionality for CDM funding.

The main limitation arises from the need to rely on available open-source data. While the UNICEF IYCF database maintains data for LMICs, breastfeeding prevalence data is lacking for many HICs, which either do not conduct the same nationally representative surveys, or do not contribute their data to the UNICEF IYCF database. This means that users need to source that data for themselves to generate GFT estimates for such countries. HIC policymakers should address the need for adequate data, to fill the gap in food system information on sustainable diets for infants and young children.

A key limitation is that the GFT does not account for the GHG impact of CMF products beyond 6 months. Follow-on formulas or growing up milk products are a large part of the CMF market and also contribute to GHG emissions comparably with infant formula; these products may account for over half of GHG emissions (37). This suggests that our GFT estimates might be approximately doubled if follow-on or growing up milk products were accounted for. Estimates based on CMF sales help address this issue but require access to expensive commercial databases.

For both practical and conceptual reasons, no adjustment is made in the GFT for the child spacing implications of breastfeeding. This is a significant limitation. Breastfeeding contributes to child spacing by decreasing fertility through lactational amenorrhea (23, 60, 61). Interventions that increase breastfeeding and decrease CMF use have positive implications for child spacing, thus considerably reducing the number of children born and thereby greatly reducing the population’s carbon footprint. An additional birth, no matter how the infant is fed, generates a lifetime of GHG emissions and other environmental impacts. One study estimates the lifetime emissions from each additional child born to be around 50 tons. However, this issue is complicated by questions about attributing intergenerational responsibilities and mitigation prospects available to future generations (62, 63). There are also huge differences in GHG impacts for a child born in a HIC compared to a LMIC, and between a child born into a rich family and a poor family within countries. Future work could examine the conceptual and measurement issues in more detail.

Also, the implications of expressing and donating human milk are not included in the GFT. Expressing milk using a breast pump generates higher GHG emissions than breastfeeding (85). Likewise, human milk banking has relatively high emissions compared to breastfeeding, due to transportation related emissions (86). However, very few infants are exclusively fed expressed breastmilk for 6 months, and infants are rarely exclusively supplied breastmilk by donor human milk banks for 6 months. In both cases, the impact of using breast pumps or supplying donor milk for short term use must also properly account for the importance of these practices as a “bridge to breastfeeding,” which avoids CMF related emissions for the older infant. The quantities involved are unlikely to be of practical significance for GFT calculations and would not be expected to alter country or global results.

Future enhancements to the GFT could include a component to calculate the carbon and water footprint for individual mothers. An automatically generated advocacy brief function that summarizes country results in a format that could motivate policy action could also be added. The option for linking the GFT to the Cost of Not Breastfeeding Tool, the Mothers’ Milk Tool, and the WBCi Costing tool to create a complete cost–benefit analysis for financing breastfeeding policies and programs (47) could also be explored. Using industry data on CMF sales would more directly and accurately measure carbon and water footprints and is incorporated into the backend GFT design: this could be considered as a valuable enhancement of the GFT if sufficient funding were available for ongoing access to such commercial data.

## 5 Conclusion

By providing additional data to incorporate into calculations of the environment impacts of not breastfeeding, the GFT allows for improved policy, programming, and budget decision making at national and global levels. It further underscores the value of interventions to support, promote and protect breastfeeding and reduce CMF consumption. Policies and investments to increase the prevalence of breastfeeding at global and country levels would reduce environmental harms by reducing demand for CMF products, which have high carbon and water footprints. Substantial reductions in CMF use could be achieved if public health interventions to protect, promote, and support breastfeeding, such as paid maternity leave, peer counseling, the International Code, and BFHI/Ten Steps, were universally in place. Furthermore, the GFT demonstrates the importance of considering breastfeeding as a fundable intervention by agencies that finance climate protection or operate carbon accounting schemes. It also creates opportunities for improved environmental impact product labeling and carbon taxes on CMF products.

The innovative GFT quantifies the environmental damage caused by demand for CMF products. By calculating the deleterious effects of CMF on the environment, as well as on human health, it fills the evidence gap for advocacy on interventions aimed at increasing exclusive breastfeeding of infants under 6 months. It is an open-source, adaptable, and user-friendly resource accessible for a variety of users, including policymakers, advocates, and researchers who work on protecting, promoting, and supporting breastfeeding. Additionally, it should encourage carbon financing schemes to consider providing funding to support breastfeeding for climate change mitigation, adaptation, and resilience.

## Ethics statement

Written informed consent was obtained from the individual(s), and minor(s)’ legal guardian/next of kin, for the publication of any potentially identifiable images or data included in this article.

## Author contributions

JS: Conceptualization, Investigation, Methodology, Validation, Writing – original draft, Writing – review & editing, Data curation, Funding acquisition, Project administration, Resources, Supervision. BB: Conceptualization, Methodology, Validation, Writing – original draft, Writing – review & editing, Formal analysis. TN: Conceptualization, Formal analysis, Methodology, Validation, Writing – original draft, Writing – review & editing, Investigation, Software, Visualization. AI: Conceptualization, Data curation, Formal analysis, Methodology, Software, Validation, Writing – review & editing. AP: Data curation, Validation, Writing – review & editing. RM: Conceptualization, Funding acquisition, Resources, Supervision, Validation, Writing – review & editing.

## Footnotes

^1^ https://nceph.anu.edu.au/research/projects/green-feeding-tool
