# Supplementary material for: Estimating carbon and water footprints associated with commercial milk formula production and use: development and implications of the Green Feeding Climate Action Tool
Source: Front Nutr. 2024 Jun 13;11:1371036. doi: 10.3389/fnut.2024.1371036 (PMC11210426; doi:10.3389/fnut.2024.1371036)
Supplement: Supplementary file 1 [file Table_1.pdf]

Supplementary Table 1

Study	Setting/ Country	Data source	Study result, tons CO2 eq. of GHG emissions, SF	GFT calculation tons CO2 eq. of GHG emissions, CMF	Validation notes	Comment
Cadwell et al (2020)	USA	Euromonitor	412,614	446,000-568,000	Considered validated, https://www.cdc.gov/breastfeeding/data/reportcard.htm	GFT gives comparable estimate
Cadwell et al (2020)	Canada	Euromonitor	2,262	32,000-40 000	Considered validated, https://health-infobase.canada.ca/breastfeeding/	GFT gives comparable estimate
Cadwell et al (2020)	Mexico	Euromonitor	59,430	167,000-213,000	Considered partly validated, https://health-infobase.canada.ca/breastfeeding/. Differences in SF and FUF definitions, and in country SF, SF and FUF feeding practices? GFT IYCF assumptions about quantities used by PBF or NBF?	GFT gives much higher estimate
Dadhich et al (2021)	Australia	Euromonitor	18,281	30,000-38,000	Considered validated at GFT emissions of 11-14 per unit of CMF, https://www.breastfeeding.asn.au/resources/breastfeeding-rates-australia	GFT gives lower estimate
Dadhich et al (2021)	China	Euromonitor	575,515	678,000-862,000	Considered partly validated at GFT emissions of 11-14 per unit of CMF, https://www.ncbi.nlm.nih.gov/pmc/articles/PMC7664678/ , https://www.oecd-ilibrary.org/sites/b2997366-en/index.html?itemId=/content/component/b2997366-en, https://internationalbreastfeedingjournal.biomedcentral.com/articles/10.1186/s13006-021-00388-y. GFT IYCF data includes institutional sales and daigou trade?	GFT gives much lower estimate
Dadhich et al (2021)	India	Euromonitor	44,621	579,000-737,000	Considered partly validated at GFT emissions of 11-14 per unit of CMF, chrome-extension: //efaidnbmnnnibpcajpcglclefindmkaj/https://dhsprogram.com/pubs/pdf/FR375/FR375.pdf	GFT gives much higher estimate

Study	Setting/ Country	Data source	Study result, tons CO2 eq. of GHG emissions, SF	GFT calculation tons CO2 eq. of GHG emissions, CMF	Validation notes	Comment
Dadhich et al (2021)	Malaysia	Euromonitor	35,945	27,000-35,000	Considered partly validated at GFT emissions of 11-14 per unit of CMF, https://www.researchgate.net/publication/320628242_Institute_for_Public_Health_IPH_2016_National_Health_and_Morbidity_Survey_2016_NHMS_2016_Maternal_and_Child_Health_Vol_I_Methodology_and_General_Findings_2016_120pp/stats	GFT gives much lower estimate
Dadhich et al (2021)	Philippines	Euromonitor	58,460	93,000-118,000	Considered validated at GFT emissions of 11-14 per unit of CMF, https://www.ncbi.nlm.nih.gov/pmc/articles/PMC9517919/#B6-ijerph-19-10938	GFT gives lower estimate
Dadhich et al (2021)	South Korea	Euromonitor	39,166	27,000-35,000	Considered partly validated at GFT emissions of 11-14 per unit of CMF, J Korean Med Sci. 2023 Aug 21;38(33):e261	GFT gives much higher estimate
Smith et al (2023)	Nepal	DHS	175,506	159000	Considered validated. Both estimates here use 14kg of GHG emissions per kg of CMF. GFT uses 20 not 21 kg per infant so will be lower. Unexplained difference maybe due to GFT estimate of CMF use by PBF infants of 6.5 kg	GFT gives lower estimate
Smith et al (2023)	Canada	Other	9,444	8890	Considered validated. Is 9334.5 if adjust to 20 kg, so rounding error?	GFT gives comparable estimate
Long et al (2021)	China (GNT savings)	Other. Data on breastfeeding rates are available internationally from UNICEF (2021), and in national studies. UNICEF data used for China reported an exclusive breastfeeding rate of 20.8% in the first five months. In this study, it is assumed that this is the exclusive breastfeeding rate for the six months under consideration. The rate of consumption will likely change	71,756	37650	Considered partly validated. GFT estimate uses 11kg not 10kg of GHG emissions per kg of CMF so increases gap. Potential CMF emissions saved from increasing EBF rate in China from 20.8% to 50% - 251,000 t CO2. Potential Irish CMF emissions saved from increasing EBF rate in China from 20.8% to 50% - 37650 t CO2	GFT gives comparable estimate

Study	Setting/ Country	Data source	Study result, tons CO2 eq. of GHG emissions, SF	GFT calculation tons CO2 eq. of GHG emissions, CMF	Validation notes	Comment
		depending on the child, so standard guidelines are used to estimate the volume consumed. There is limited information on partial or mixed feeding, so this is not included in the study. Ireland provides 15 percent of the imported infant food and formula in China				
Long et al (2021)	Ireland (GNT savings)	Other. Data on breastfeeding rates are available internationally from UNICEF (2021), and in national studies. The most current Irish information available came from the ESRI Growing up in Ireland longitudinal study (Layte and McCrory, 2014) which reported an initial exclusive breastfeeding rate of approximately 30% in month one that decreases to 2% by month six. For the Irish scenario, the volume of breast-milk substitutes consumed per infant is estimated and multiplied by the annual birth rate. The rate of consumption will likely change depending on the child, so standard guidelines are used to estimate the volume consumed. There is limited information on partial or mixed feeding, so this is not included in the study.	4,963	5864	Considered validated. GFT uses 11 kk CO2 eq per kg of CMF compared to 10 in Long et al https://www.ncbi.nlm.nih.gov/pmc/articles/PMC5057437/ https://www.hse.ie/eng/about/who/healthwellbeing/our-priority-programmes/child-health-and-wellbeing/breastfeeding-healthy-childhood-programme/research-and-reports-breastfeeding/breastfeeding-on-the-island-of-ireland-report.pdf Layte, R. McCrory, C. (2014). Maternal Health Behaviours and Child Growth in Infancy: Analyses of the Infant Cohort of the Growing Up in Ireland Study. Dublin: The Stationery Office. available at chrome-extension://efaidnbmnnnibpcajpcglclefindmkaj/https://www.drugsandalcohol.ie/23285/1/Infant_Maternal_Health_4_web.pdf Potential emissions saved from achieving 50% EBF rate 5864 t CO2	GFT gives comparable estimate

Study	Setting/ Country	Data source	Study result, tons CO2 eq. of GHG emissions, SF	GFT calculation tons CO2 eq. of GHG emissions, CMF	Validation notes	Comment
Joffe et al (2019)	UK (support savings)	Euromonitor	50000-77500 (car miles)	28261 (car miles)	Considered partly validated. Study provides insufficient births or infant feeding data to replicate, approximation of EBF/PBF and NBF based on Cleminson J, et al. Arch Dis Child Fetal Neonatal Ed 2015;100:F173–F178. doi:10.1136/archdischild-2013-304873 127-162 million kg, equivalent to 28 261 cars off the road if all mothers giving birth in a year were supported to breastfeed exclusively for 6 months. 127-162 million kg, equivalent to 28 261 cars off the road if all mothers giving birth in a year were supported to breastfeed exclusively for 6 months	GFT gives lower estimate
